# Antibiotic therapy completion for injection drug use-associated infective endocarditis at a center with routine addiction medicine consultation: a retrospective cohort study

**DOI:** 10.1186/s12879-022-07122-x

**Published:** 2022-02-05

**Authors:** Muhammad Dhanani, Courtney Goodrich, Janice Weinberg, Carlos Acuna-Villaorduna, Tamar F. Barlam

**Affiliations:** 1 Section of Infectious Diseases, Department of Medicine, Boston Medical Center, 801 Massachusetts Avenue 2nd Fl., Boston, MA 02118 USA; 2 Department of Biostatistics, School of Public Health, Boston University, 801 Massachusetts Avenue 3rd Fl., Boston, MA 02118 USA; 3 Evans Center for Interdisciplinary Biomedical Research, Boston University, 700 Albany Street W601, Boston, MA 02118 USA; 4 Graduate Medical Sciences, Boston University School of Medicine, 72 East Concord Street, Boston, MA 02118 USA; 5 Section of Infectious Diseases, Lemuel Shattuck Hospital, 170 Morton Street, Boston, MA 02130 USA; 6 Section of Infectious Diseases, Department of Medicine, Boston University School of Medicine, 801 Massachusetts Avenue 2nd Fl., Boston, MA 02118 USA; 7 Present Address: Division of Infectious Disease, Department of Medicine, Northwestern University, 645 N. Michigan Avenue Rm. 929, Chicago, IL 60611 USA

**Keywords:** Endocarditis, Injection drug use, Opioid-related disorders, Medications for opioid use disorder

## Abstract

**Background:**

Addiction medicine consultation and medications for opioid use disorder are shown to improve outcomes for patients hospitalized with infective endocarditis associated with injection drug use. Existing studies describe settings where addiction medicine consultation and initiation of medications for opioid use disorder are not commonplace, and rates of antibiotic therapy completion are infrequently reported. This retrospective study sought to quantify antibiotic completion outcomes in a setting where these interventions are routinely implemented.

**Methods:**

Medical records of patients hospitalized with a diagnosis of bacteremia or infective endocarditis at an urban hospital between October 1, 2015 and December 31, 2017 were screened for active injection drug use within 6 months of hospitalization and infective endocarditis. Demographic and clinical parameters, receipt of antibiotics and medications for opioid use disorder, and details of re-hospitalizations within 1 year of discharge were recorded.

**Results:**

Of 567 subjects screened for inclusion, 47 had infective endocarditis and active injection drug use. Addiction medicine consultation was completed for 41 patients (87.2%) and 23 (48.9%) received medications for opioid use disorder for the entire index admission. Forty-three patients (91.5%) survived to discharge, of which 28 (59.6%) completed antibiotic therapy. Twenty-nine survivors (67.4%) were re-hospitalized within 1 year due to infectious complications of injection drug use.

**Conclusions:**

Among patients admitted to a center with routine addiction medicine consultation and initiation of medications for opioid use disorder, early truncation of antibiotic therapy and re-hospitalization were commonly observed.

**Supplementary Information:**

The online version contains supplementary material available at 10.1186/s12879-022-07122-x.

## Background

The opioid crisis is a pressing public health concern. In 2018, an estimated 11 million Americans had opioid use disorder (OUD) [1]. Both opioid-related deaths due to overdose and infectious complications of injection drug use (IDU) have been rising in incidence. Strategies to address injection drug use-associated infective endocarditis (IDU-IE) are urgently needed due to its high morbidity and significant healthcare costs [2–4].

Use of medications for OUD (MOUD) and involvement of addiction medicine specialists improve outcomes for patients with OUD and infectious complications of IDU when implemented at sites where these interventions are infrequent. In one study, involvement of addiction medicine specialists improved antibiotic therapy completion rates and prolonged time to re-hospitalization [5]. However, treatment outcomes at sites where these interventions are routinely implemented are unknown.

We sought to examine outcomes among patients admitted for treatment of IDU-IE at a site experienced in the care of hospitalized patients with OUD, where addiction medicine consultation [6] is routinely sought and use of MOUD while patients are hospitalized is commonplace. Rates of antibiotic therapy completion and re-hospitalization due to IDU-related illness were examined.

## Methods

All patients hospitalized with a diagnosis of bacteremia or infective endocarditis (IE) between October 1, 2015 and December 31, 2017 at a tertiary center were reviewed. The beginning of the date range reflects the first day where hospital billing required use of the *International Classification of Diseases, Tenth Revision* (ICD-10). The closing date was selected to ensure adequate follow-up data. The addiction medicine consultation service was formed in July 2015, and so it was available for clinical care for all patients who would be otherwise eligible for inclusion. The Institutional Review Board at Boston University School of Medicine approved all study protocols.

Eligible individuals were identified using discharge ICD-10 codes for bacteremia and IE (B37.6, I33, I76 and R78.81). Charts were reviewed by a single author. Charts were reviewed to identify active IDU within 6 months of hospitalization, as documented by providers or as supported by urine toxicology assays and physical examination findings. The presence of suspected or definite IE was confirmed using the modified Duke criteria [7]. Exclusion criteria consisted of pregnancy, absence of IDU or bacteremia, and diagnosis of IE at an external facility within 6 months of hospitalization.

Further chart review captured demographics, receipt of antibiotics and MOUD, involvement of addiction medicine and infectious diseases (ID) specialists, and other clinical information including subsequent hospitalizations. Medical records at other hospitals accessible through links integrated into the electronic health record were reviewed. IDU-related infections were defined to include bacteremia, IE, osteomyelitis, septic arthritis and skin & soft tissue infections.

Overall rates of IDU-IE antibiotic therapy completion were calculated. Patients were considered to have completed IDU-IE therapy if they completed the initial regimen or a regimen that had been adjusted by an ID specialist. Interruptions were permitted if less than 72 h in duration. Rates of readmission for treatment of IDU-related infections were also calculated. Descriptive statistics were calculated using SAS v9.4 (Cary, N.C.).

## Results

**Fig. 1:**
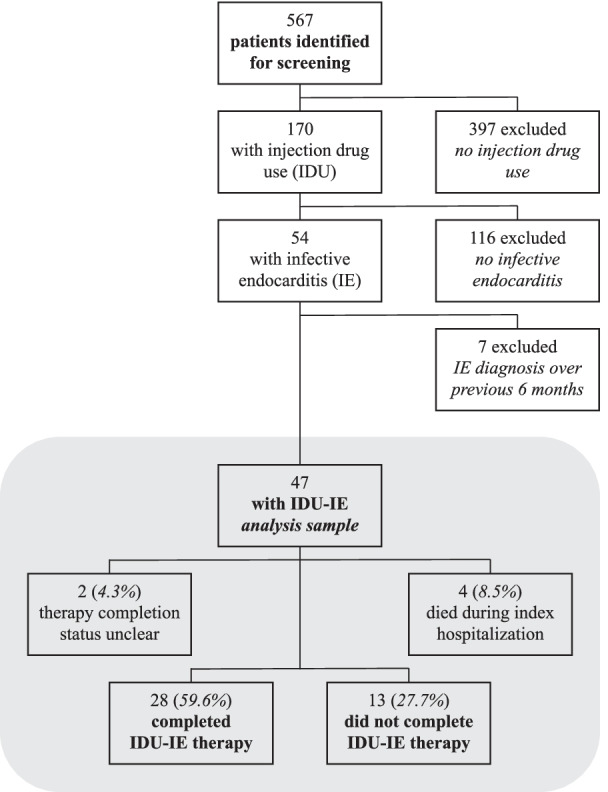
Reasons for exclusion and therapy completion outcomes

Initially, 567 subjects were screened for eligibility; 397 lacked evidence of IDU and were excluded. Of the remaining 170 patients, 116 had evidence of IDU but not IDU-IE, and 7 had received a diagnosis of IDU-IE elsewhere. Ultimately, 47 patients comprised the cohort (Fig. 1).

The median age of the patients with IDU-IE was 37.0 years (interquartile range, 28.0 to 48.0) and 55.3% were men. The self-reported racial distribution was 53.2% non-Hispanic white and 27.7% African-American. Twenty-two subjects (46.8%) were identified as having housing instability (Table 1). Thirty-seven patients (78.7%) had definite IE while 10 (21.3%) had suspected IE. All subjects had OUD and specifically injected opioids while 41 (87.2%) had coexisting cocaine use disorder. All patients received ID consultation, and 41 (87.2%) were evaluated by an addiction medicine specialist. Forty-two patients (89.4%) received MOUD at least once, and 23 (48.9%) received MOUD for their entire admission. Four patients (8.5%) died. In terms of MOUD agents utilized, methadone was used in 37 patients (78.7%), and buprenorphine/naloxone was used in 2 patients (4.3%). Details of substance use disorder (SUD) diagnoses and MOUD are presented in Additional file 1. Details of IDU-IE are presented in Additional file 2.

**Table 1:** Baseline characteristics of infective endocarditis cohort (*N* = 47)

Characteristic	*N*	Percentage (%)
Age, median (IQR), y	37.0 (28.0–48.0)	
Male sex	26	55.3
Race		
White	25	53.2
African-American	13	27.7
Declined/Not Available	9	19.1
Housing instability	22	46.8
AMA discharge within 1 year	14	29.8
Comorbidities		
Diabetes mellitus	4	8.5
Known prior IE	7	14.9
Prosthetic cardiac valve in situ	2	4.3
HIV seropositive	2	4.3
HCV seropositive	37	78.7
Length of stay, median (IQR), d	17.0 (9.0–33.0)	
Duration of bacteremia, median (IQR), d	5.0 (3.0–7.0)	
Required ICU care	24	51.1
Infectious diseases consultation	47	100
Addiction medicine consultation	41	87.2
Surgery during admission	9	19.1
Cardiac valve replacement surgery during admission	5	10.6
Discharge destination		
Home	5	10.6
Urban state hospital	19	40.4
Suburban state hospital	2	4.3
Other subacute facility	9	19.1
Discharged AMA	8	17.0
Died	4	8.5

AMA, Against medical advice; HCV, Hepatitis C; HIV, Human immunodeficiency virus; ICU, Intensive care unit; IE, Infective endocarditis; IQR, Interquartile range

Among the 43 survivors, 28 patients (65.1%) completed the original regimen or one that was adjusted by an ID specialist. In 13 patients (30.2%), antibiotic therapy was truncated due to an against medical advice departure in the acute or postacute setting. The outcome was unknown for 2 patients (4.7%) (Table 2). As detailed in Additional file 3, 38 (88.4%) were re-hospitalized for treatment of an infection within 1 year of discharge, 29 (67.4%) of whom had an IDU-related infection such as bacteremia or a skin & soft tissue infection. Among this group, 1 patient was found to have IE from the previously-identified pathogen while 6 were found to have IE due to a different pathogen. All 28 survivors discharged when medically indicated were provided a referral to an outpatient MOUD program that had agreed to provide ongoing management for them.

**Table 2:** Outcomes among Survivors of Infective Endocarditis Cohort (*N* = 43)

Characteristic	*N*	Percentage (%)
Antibiotic regimen outcome		
Completed antibiotic regimen	28	65.1
Without discharge from acute setting	4	9.3
With non-AMA discharge from acute setting	18	41.9
With AMA-related interruption < 72 h	2	4.7
Regimen altered or shortened by specialist after discharge	4	9.3
Truncated regimen due to non-medical cause (e.g., AMA departure)	13	30.2
Outcome unknown	2	4.7
Readmission		
Readmitted within 1 year of discharge	38	88.4
> 1 Readmission within 1 year of discharge	25	58.1
Readmission to an EHR-linked facility	10	23.3
Readmission for infection unrelated to IDU	29	67.4
Readmission for infection unlikely to be IDU-related	7	16.3

AMA, against medical advice; EHR, Electronic health record; IDU, Injection drug use

## Discussion

Ultimately, 65.1% of our cohort's survivors completed antibiotic therapy, 87.2% were seen by addiction medicine specialists and 89.4% received MOUD. This rate of MOUD initiation differs greatly from the initiation rate of 5.7% reported in a nationwide study of IDU-IE patients [8]. Antibiotic therapy completion in IDU-IE has not been studied extensively; one study of 26 patients reported a completion rate of 92.3% [9]. In a study of patients hospitalized with infectious complications of IDU including but not limited to IDU-IE, 52.0% completed antibiotic therapy, and 30.4% received addiction medicine consultation [5]. Notably, 38.4% of patients in that study received MOUD, whereas 48.9% of patients in our study received it for their entire hospitalization. Thus, a sizeable fraction of patients in both cohorts did not complete antibiotic therapy despite relatively higher use of SUD-oriented interventions at our facility.

In the cohort of Marks et al. [5], 113 IDU-IE patients were included. An overall 90-day readmission rate of 36.3% was found; among those with and without addiction medicine evaluation, this rate was 28.6% and 54.5%, respectively (L. R. Marks, personal communication, June 5, 2020). In our study, a longer follow-up period was examined, and 88.4% of patients were re-hospitalized at least once. Thus, at our center with its widespread utilization of SUD-directed interventions, readmissions were common. MOUD may be just one critical component of improving outcomes among this population; others have called for broader awareness of social determinants of health, stating that medical interventions represent just one aspect in optimizing the care of this vulnerable patient population [10].

Transitions of care are a key consideration in evaluating the outcomes observed in our study, since most survivors in our cohort were discharged to subacute facilities. Federal policy, specifically Title 21 of the Code of Federal Regulations, complicates patients’ receipt of MOUD at these facilities, preventing continuation of MOUD started in the inpatient setting unless the patient is already enrolled in an OUD treatment program [11]. While facilities were expected to continue appropriate therapies after discharge, we were unable to independently verify continuation of MOUD after discharge. Strategies to improve outcomes among those transitioning to a non-facility setting have also been studied. In one randomized trial of patients with an IDU-related infection, patients in the experimental arm underwent frequent outpatient visits following an inpatient stay rather than remaining hospitalized for antibiotic therapy alone; all completed antibiotic therapy [12], suggesting careful planning after discharge can improve antibiotic therapy completion. SUD-specific care management teams have been proposed [13], and may provide key support while patients transition to the outpatient setting.

The major limitation of our study is its sample size, which limited the ability to perform analytical statistics beyond descriptive calculations. Although not all patients were seen by the addiction medicine service, dichotomizing the sample by that factor created subgroups that were themselves too small for an analytic approach. Similarly, the absence of a control group also prevented us from taking an analytical approach. The boundaries of the eligibility window, which determined the study size, were chosen to allow for exclusive use of ICD-10 billing codes while leaving sufficient time for 12 months of follow up. ICD-10 codes were only used to capture infection-related diagnoses, and not to identify SUDs, for which they are unreliable [14, 15]. In addition, the study eligibility window accounted for the availability of addiction medicine consultation, and allowed study results to reflect contemporary issues in the treatment of IDU-IE. This is particularly pertinent because fentanyl increasingly replaced heroin to become the dominant opioid in our region over the period of the study [16]. Other key limitations were the inability to assess engagement in SUD-specific care and receipt of MOUD after hospital discharge. While the availability of post-discharge MOUD receipt data would have added greatly to the study's impact, these data were not available as described above. Additionally, follow up data were not available for two individuals. A final limitation of our study is that it was performed at a single institution that largely cares for underserved and low-income patients, and so its generalizability is limited.

## Conclusions

In settings where addiction medicine expertise is routinely integrated into the care of patients hospitalized with IDU-IE, completion of antibiotic therapy may remain suboptimal and re-hospitalization for IDU-related illness may be commonplace. Attention to transitions of care and sustained support from SUD-specific services will likely prove essential to improving these outcomes.

## Supplementary Information

**Additional file 1:** Substance Use Disorder & Treatment Details. This file contains a table that provides further details regarding the specific substance use disorders reported by members of the cohort, and the types of MOUD prescribed to them.

**Additional file 2:** Infective Endocarditis Details. This file contains a table that provides further details regarding the laterality, complications and microbiology of the infective endocarditis diagnoses described by the study.

**Additional file 3:** Readmissions for Infectious Sequelae of Injection Drug Use. This file contains a table that depicts reasons for re-hospitalization among members of the cohort.

## Data Availability

The datasets generated and analysed during the current study are not publicly available due to property rights of the sponsoring hospital, but are available from the corresponding author on reasonable request.

## Abbreviations

AMA: Against medical advice
EHR: Electronic health record
HCV: Hepatitis C
HIV: Human immunodeficiency virus
ICD-10: *International Classification of Diseases, 10th Revision*
ICU: Intensive care unit
ID: Infectious diseases
IDU: Injection drug use
IDU-IE: Injection drug use-associated infective endocarditis
IE: Infective endocarditis
IQR: Interquartile range
OUD: Opioid use disorder
SUD: Substance use disorder
