# Supplementary material for: Antibiotic therapy completion for injection drug use-associated infective endocarditis at a center with routine addiction medicine consultation: a retrospective cohort study
Source: BMC Infect Dis. 2022 Feb 5;22:128. doi: 10.1186/s12879-022-07122-x (PMC8818134; doi:10.1186/s12879-022-07122-x)
Supplement: Supplementary file 1 — Additional file 1: Substance Use Disorder & Treatment Details. This file contains a table that provides further details regarding the specific substance use disorders reported by members of the cohort, and the types of MOUD prescribed to them. [file 12879_2022_7122_MOESM1_ESM.docx]

Additional File 1: Substance Use Disorder & Treatment Details (*N* = 47)

| **Characteristic** | ***N*** | **Percentage** |
| --- | --- | --- |
| Substance use disorder*  Benzodiazepine + opiate  Cocaine + opiate  Benzodiazepine + cocaine + opiate  Opiate alone  Duration of IDU  Between than 1 and 5 years  Between 5 and 10 years  Greater than 10 years  Not assessed  Type of MOUD received  methadone  buprenorphine/naloxone  mixture  Received MOUD of any kind  < 75% of hospital days  ≥ 75% to < 90% of hospital days  ≥ 90% to < 100% of hospital days  100% of hospital days | 13  41  11  4  5  9  23  10  37  2  3  42  11  1  7  23 | 27.7%  87.2%  23.4%  8.5%  10.6%  19.1%  48.9%  21.3%  78.7%  4.3%  6.4%  89.4%  23.4%  2.1%  14.9%  48.9% |

*Cocaine use was not assessed in 2 patients (4.3%); benzodiazepine use was not assessed in 5 patients (10.6%)

Abbreviations: Injection drug use (IDU); Medication for opioid use disorder (MOUD).
