# Supplementary material for: Antibiotic therapy completion for injection drug use-associated infective endocarditis at a center with routine addiction medicine consultation: a retrospective cohort study
Source: BMC Infect Dis. 2022 Feb 5;22:128. doi: 10.1186/s12879-022-07122-x (PMC8818134; doi:10.1186/s12879-022-07122-x)
Supplement: Supplementary file 2 — Additional file 2: Infective Endocarditis Details. This file contains a table that provides further details regarding the laterality, complications and microbiology of the infective endocarditis diagnoses described by the study. [file 12879_2022_7122_MOESM2_ESM.docx]

Additional File 2: Infective Endocarditis Details (*N* = 47)

| **Characteristic** | ***N*** | **Percentage** |
| --- | --- | --- |
| IE classification  Suspected  Definite  Disease complications  Uncomplicated  Visceral embolization  Septic pulmonary embolization only  Incomplete radiology evaluation  Vegetation presence  No vegetation identified  Aortic  Mitral  Tricuspid  Aortic + tricuspid  Mitral + tricuspid  Incomplete echocardiography evaluation  Organism  Gram-negative  MSSA  MRSA  viridans group *Streptococci*  Other monomicrobial  Polymicrobial | 10  37  1  22  18  6  13  5  9  17  2  1  4  1  18  18  4  3  3 | 21.3%  78.7%  2.1%  46.8%  38.3%  12.8%  27.7%  10.6%  19.1%  36.2%  4.3%  2.1%  8.5%  2.1%  38.3%  38.3%  8.5%  6.4%  6.4% |

Abbreviations: Infective endocarditis (IE); Methicillin-sensitive *Staphylococcus aureus* (MSSA); Methicillin-resistant *Staphylococcus aureus* (MRSA)
