# Supplementary material for: Antibiotic therapy completion for injection drug use-associated infective endocarditis at a center with routine addiction medicine consultation: a retrospective cohort study
Source: BMC Infect Dis. 2022 Feb 5;22:128. doi: 10.1186/s12879-022-07122-x (PMC8818134; doi:10.1186/s12879-022-07122-x)
Supplement: Supplementary file 3 — Additional file 3: Readmissions for Infectious Sequelae of Injection Drug Use. This file contains a table that depicts reasons for re-hospitalization among members of the cohort. [file 12879_2022_7122_MOESM3_ESM.docx]

Additional File 3: Readmissions for Infectious Sequelae of Injection Drug Use (*N* = 43)

| **Characteristic** | ***N*** | **Percentage** |
| --- | --- | --- |
| Any readmission for infection related to IDU  Skin & soft tissue infection or abscess only  Osteomyelitis or septic arthritis  Bacteremia distinct from index organism  IDU-IE distinct from index organism  Bacteremia identical to index organism  IDU-IE identical to index organism | 29  9  4  8  6  5  1 | 67.4%  20.9%  9.3%  18.6%  14.0%  11.6%  2.3% |

Abbreviations: Injection drug use (IDU); Injection drug use-associated infective endocarditis (IDU-IE).
